# Global Analyses of Expressed Piwi-Interacting RNAs in Gastric Cancer

**DOI:** 10.3390/ijms21207656

**Published:** 2020-10-16

**Authors:** Tatiana Vinasco-Sandoval, Fabiano Cordeiro Moreira, Amanda F. Vidal, Pablo Pinto, André M. Ribeiro-dos-Santos, Rebecca L. S. Cruz, Gleyce Fonseca Cabral, Ana K. M. Anaissi, Katia de Paiva Lopes, Arthur Ribeiro-dos-Santos, Samia Demachki, Paulo Pimentel de Assumpção, Ândrea Ribeiro-dos-Santos, Sidney Santos

**Affiliations:** 1 Graduate Program in Genetics and Molecular Biology, Laboratory of Human and Medical Genetics, Federal University of Pará, Belém, Pará 66075-110, Brazil; gloria.vinasco-sandoval@cea.fr (T.V.-S.); fabiano.ufpa@gmail.com (F.C.M.); amandaferreiravidal@gmail.com (A.F.V.); pablopintonet@yahoo.com (P.P.); andremrsantos@gmail.com (A.M.R.-d.-S.); rebeccalais94@gmail.com (R.L.S.C.); cabralffg@gmail.com (G.F.C.); katiaplopes@gmail.com (K.d.P.L.); arthurrdsantos@outlook.com (A.R.-d.-S.); akelyufpa@gmail.com (Â.R.-d.-S.); 2 Graduate Program in Oncology and Medical Sciences, Center of Oncology Research, Federal University of Pará, Belém, Pará 66063-023, Brazil; anakaryssa@yahoo.com (A.K.M.A.); demachki@gmail.com (S.D.); assumpcaopp@gmail.com (P.P.d.A.)

**Keywords:** PIWI-interacting RNA (piRNA), gastric cancer, piRNAs expression profile (piRNome), field cancerization, field-effect, cancer biomarkers, metastasis, non-coding RNA

## Abstract

Gastric cancer (GC) represents a notable amount of morbidity and mortality worldwide. Understanding the molecular basis of CG will offer insight into its pathogenesis in an attempt to identify new molecular biomarkers to early diagnose this disease. Therefore, studies involving small non-coding RNAs have been widely explored. Among these, PIWI-interacting RNAs (piRNAs) are an emergent class that can play important roles in carcinogenesis. In this study, small-RNA sequencing was used to identify the global piRNAs expression profile (piRNome) of gastric cancer patients. We found 698 piRNAs in gastric tissues, 14 of which were differentially expressed (DE) between gastric cancer (GC), adjacent to gastric cancer (ADJ), and non-cancer tissues (NC). Moreover, three of these DE piRNAs (piR-48966*, piR-49145, piR-31335*) were differently expressed in both GC and ADJ samples in comparison to NC samples, indicating that the tumor-adjacent tissue was molecularly altered and should not be considered as a normal control. These three piRNAs are potential risk biomarkers for GC, especially piR-48966* and piR-31335*. Furthermore, an in-silico search for mRNAs targeted by the differentially expressed piRNAs revealed that these piRNAs may regulate genes that participate in cancer-related pathways, suggesting that these small non-coding RNAs may be directly and indirectly involved in gastric carcinogenesis.

## 1. Introduction

Gastric cancer (GC) is the fifth most common malignancy in the world and the third leading cause of cancer-related death worldwide [1]. Due to the lack of specific symptoms, most gastric cancer patients are diagnosed in advanced-stage disease with a poor prognosis [2,3]. Unfortunately, the molecular mechanism of gastric cancer formation and progression has not been completely elucidated [4,5].

GC exhibits a wide range of molecular alterations, including pathways involved in mitosis, immune signaling, cell adhesion, and migration [6,7]. These alterations have been described both in genetic and epigenetic levels [8,9] and many studies have deeply investigated these epigenetic alterations [10,11]. Particularly, the function of non-coding RNAs (ncRNAs) in cancer development and progression [12,13,14], mostly the role of miRNAs in post-transcriptional regulation, has been very well characterized [12,15,16].

PIWI-interacting RNAs (piRNAs) are a type of small ncRNAs of 26 to 31 nucleotides that act as a sequence-specific guide for PIWI proteins and have been shown to be implicated in the maintenance of genome integrity by acting as methylation regulators and gene controllers at transcriptional and posttranscriptional levels [17,18,19,20].

There are more than 30,000 piRNA isoforms described in the human genome, and it is believed that these molecules compose the most abundant and diverse regulatory noncoding RNA class [21,22,23]. Although for several years the functions to piRNAs were focused only in the genome integrity and development of germinal stem cells [24,25,26], the functions of piRNAs and PIWI proteins as epigenetic regulators have started to emerge, and a subset of piRNAs has also been implicated in the regulation of protein-coding genes [27,28,29,30].

Several studies have shown that the piRNAs are broadly expressed in germline cells. More recently, it has been described by high throughput sequencing that their signaling pathway is also active in somatic cells, especially in human cancers [30,31,32,33,34], suggesting their possible role in carcinogenesis. So far, piRNAs have been associated with some hallmarks of cancer, such as sustaining proliferative signaling, evading growth suppressors, activating invasion, and metastasis [35,36,37,38]. Furthermore, some cancer-associated piRNAs were found in a patient’s bloodstream [39], meaning that piRNAs may be potential biomarkers of the disease, although there are some discrepancies.

However, their involvement in gastric carcinogenesis is still poorly investigated and more studies are needed in attempting to elucidate it. This study aimed to investigate and compare the expression profile of piRNAs in gastric samples from patients without cancer, gastric cancer samples, and samples from the matched tumor-adjacent tissue and, moreover, to identify potential piRNA biomarkers of GC.

## 2. Results

**Figure 1:**
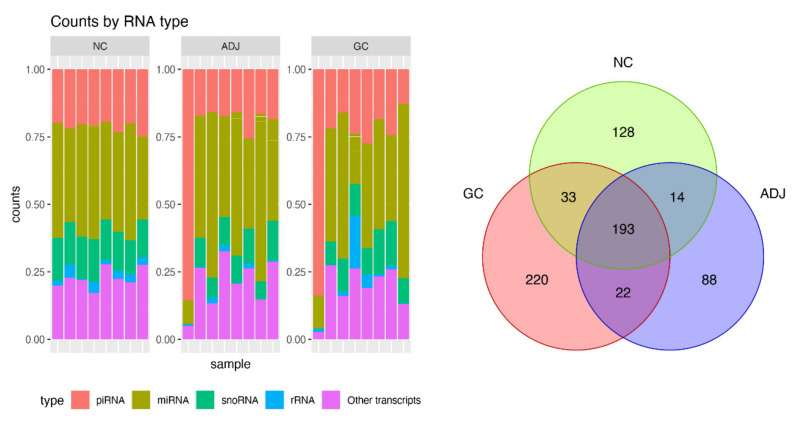
Summary of small RNA sequencing data. Barplot of percent of small non-coding RNA (sncRNA) transcripts identified per sample (left panel) and Venn diagram for number of PIWI-interacting RNAs (piRNAs) detected by small RNA-Seq in three groups of gastric tissue samples. Gastric cancer (CG), matched tumor-adjacent gastric tissue (ADJ) and non-cancer (NC) (right panel).

A high throughput small RNA-sequencing experiment was conducted in 24 gastric tissue samples. After trimming the adapters, quality control, alignment to human genome (hg19), and transcripts quantitation, we obtained, on average, 1.2 million reads per sample. Several small Non-coding RNAs and other transcripts fragments were identified (Figure 1—left panel) and from them, about 20% (~5 million reads) were recognized as piRNA reads, representing 698 different piRNAs. The global statistics on the reads are presented in Supplementary Materials Table S1. 

Approximately 90% of recognized piRNAs had between 30 and 35 bp (Supplementary Figure S1), and 83% of the piRNAs reads were concentrated on the 20 most expressed piRNAs. As shown in Figure 1 (right panel), there were 193 piRNAs expressed in all sample groups and several that were group-exclusive.

### 2.1. Differential Expression Analysis

**Figure 2:**
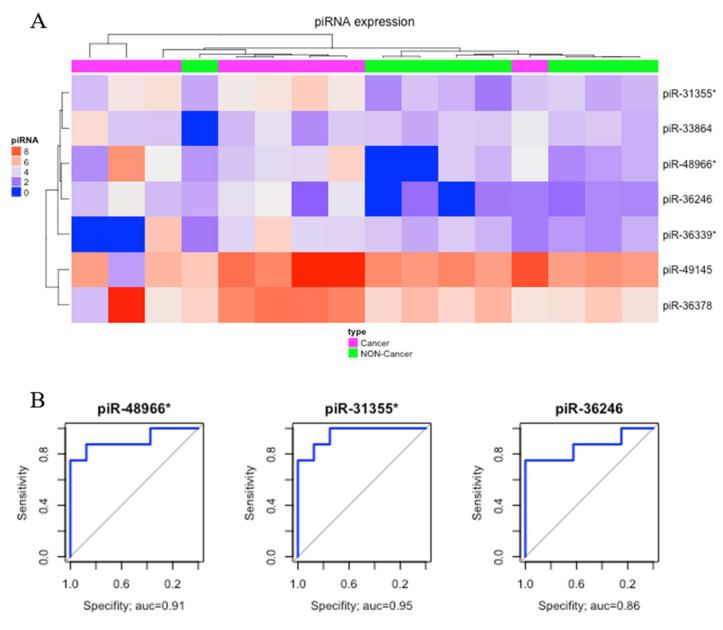
Differentially expressed piRNAs between gastric cancer and non-cancer. (**A**) Hierarchic clustering heat map of differentially expressed piRNAs (*p*-value < 0.05; |fold-change| > 3). (**B**) The receiver operating characteristic (ROC) curve analysis of piRNAs that presented AUC > 0.85.

Comparing gastric cancer (GC) samples with gastric non-cancer (NC) samples, we found seven significantly upregulated piRNAs (Table 1). The heatmap obtained from this comparison is presented in Figure 2A. Three of these differentially expressed (DE) piRNAs (piR-48966*, piR31355*, and piR36246) showed AUC > 0.85 and were selected as potential biomarkers of gastric cancer (Figure 2B).

**Figure 3:**
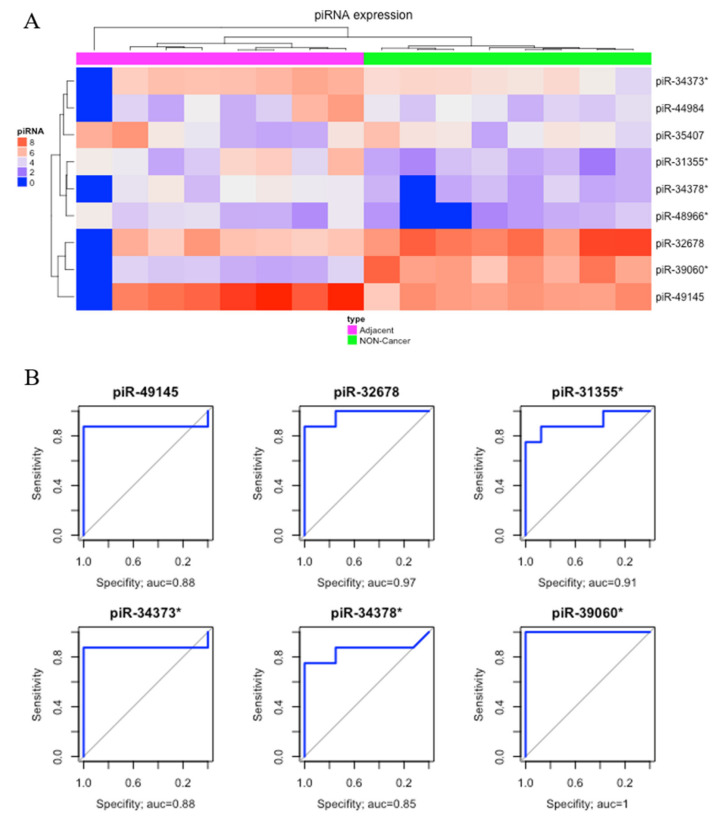
Differentially expressed piRNAs between tumor-adjacent gastric tissue and non-cancer. (**A**) Hierarchical clustering heat map of differentially expressed piRNAs (*p*-value < 0.05; |fold-change| > 3). (**B**) The ROC analysis of differentially expressed piRNAs identified potential earlier gastric cancer biomarkers with the best sensibility/specificity relation (AUC > 0.85).

Comparing matched tumor-adjacent gastric tissue samples (ADJ) with NC, we found nine DE piRNAs, two of which were down-regulated and seven were up-regulated (Table 1). The heatmap obtained from this comparison was able to perfectly cluster ADJ and NC samples (Figure 3A). Six of these DE piRNAs (four up-regulated, piR-49145, piR-31355*, piR-34373*, and piR-34378*; and two down-regulated, piR-39060* and piR-32678) had the best sensibility/specificity relationship (AUC > 0.85) and were selected as potential biomarkers to allow early diagnosis of GC (Figure 3B).

Since sample ADJ.100927_1B (first sample from left to right in Figure 3A) presented a different expression pattern, which might have been caused by technical issues, we double-checked the sample features, realized that it had a reasonable read count, and found that the identified pattern could also be related to a biological peculiarity. Aiming to verify the consequences of removing the sample, we re-analyzed the clustering steps, and no significant modification of results among the groups was found (Supplementary Figures S2 and S3).

Comparing GC with ADJ, we found three piRNAs (piR-36378, piR-33534*, and piR-39060*) that were significantly upregulated in GC (Table 1). However, the hierarchic clustering was not able to cluster GC and ADJ samples. The piRNA piR-39060* was also differentially expressed in the ADJ vs. NC analysis. piR-33534* and piR-36378 were exclusively differentially expressed in the GC vs. ADJ analysis, although none of these DE piRNAs presented high sensibility/specificity relation in the receiver operating characteristic (ROC) analysis (AUC < 0.85).

**Figure 4:**
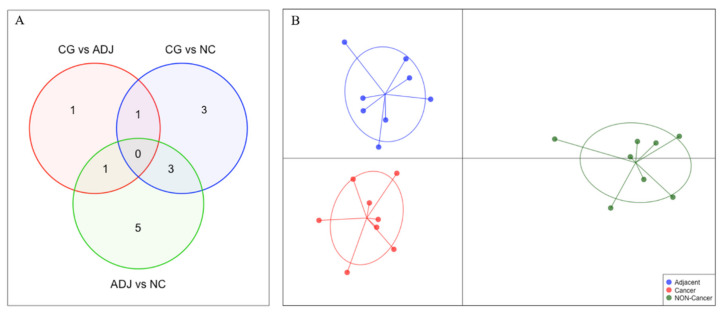
Comparison of the differentially expressed piRNAs (|fold-change| > 3 and *p*-value < 0.05) among all three analyses. (**A**) Venn Diagram identifying the number of differentially expressed piRNAs in each comparison. (**B**) Discriminant analysis of principal components (DAPC) plot clustering samples based on differentially expressed piRNAs.

The small number of DE piRNAs in GC vs. ADJ suggests that these tissues have similar piRNA expression profiles. Using the discriminant analysis of principal components (DAPC) plot with all DE piRNAs, we observed that GC, ADJ, and NC samples generated distinct clusters, indicating expression differences among these tissues (Figure 4).

Table 1 data unveiled a group of piRNAs similarly altered in both GC and ADJ samples compared to NC samples, which may be involved in the process of field cancerization in the stomach (piR-48966* piR-49145 and piR-31355*).

**Figure 5:**
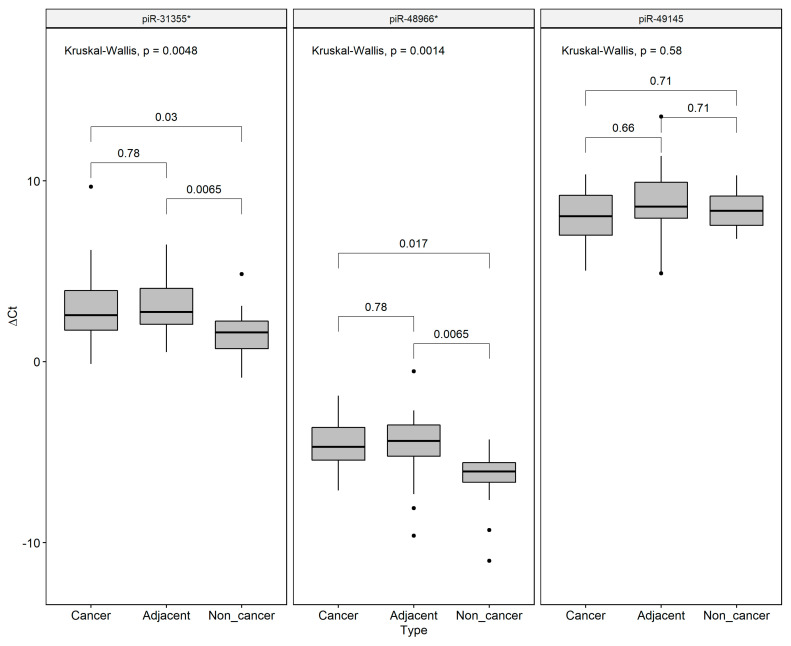
Expression values (*∆Ct*) of the field effect-related piRNAs (piR-48966* piR-49145 and piR-31355*) in gastric cancer, matched tumor-adjacent gastric tissue, and non-cancer samples, respectively. U6 Small Nuclear RNA (RNU6) was used as an endogenous control. *p-values were adjusted to multiple comparisons by Bonferroni’s correction. Individual black points (•) are outliers.

We decided to validate the field effect-related piRNAs by qRT-PCR, revealing that piR-31355* was overexpressed in both GC and ADJ when compared to NC samples, corroborating our RNA-Seq results. We also found a significant difference in piR-48966* expression between ADJ and NC samples. No difference was found for pir-49145 expression among the groups (Figure 5).

### 2.2. Identification of the Cancer-Related piRNAs Differentially Expressed Target Genes

We performed two approaches to identify target genes of the differentially expressed piRNAs. First, considering that mature piRNAs in cytoplasm form a complex with PIWI proteins and migrate back into the nucleus, reaching their target transcripts and mobilizing the silencing machinery to block the transcription of DNA, we performed a BLAST search using a mature piRNA sequence as a query. We identified 33 genes that exhibited at least 13 contiguous nucleotides complementary matches (supplementary Table S3). Among these 33 genes found, we found eight cancer-related genes, which seem to be regulated by five DE piRNAs. 

We used a Miranda tool to predict biologically relevant RNA–RNA interactions between each DE piRNA and the cancer-related genes. Interestingly, we found more than one match between the DE piRNAs and their target genes in most of the cases (Table 2), suggesting that these genes may be regulated by the pi-RISC complex at the transcriptional level. Using RNA-Seq and small RNA-Seq data from 64 paired tissues from ENCODE, we correlated the expression of DE piRNAs and their possible target genes, revealing a negative correlation between *PIKFYVE* and piR-31355* (*r* = −0.27, *p* = 0.028) (Supplementary Figure S4).

Second, to identify the possible targets of the DE piRNAs at the post-transcriptional level, we considered that piRNAs can form specific RNA silencing complexes (pi-RISC) capable of promoting mRNA repression via imperfect base-pairing between the two RNAs, by a mechanism that closely resembles the regulation by miRNAs. Thus, we searched, within UTR3′, UTR5′, and CDS transcript databases, all RNAs that, according to this mechanism, may be potential targets for the 14 DE piRNAs. We found 2248 RNA–RNA interactions between piRNA and mRNAs that corresponded to 967 genes from the human genome. To each piRNA, we found a complementarity to range from 2 to 252 genes (Table 3).

We performed the functional analysis by grouping all post-transcriptional target genes of the DE piRNAs. This approach revealed that several proteins encoded by these putative piRNA-target mRNAs are involved in key cellular processes in cancer, including cell adhesion, signaling and interaction, and cell morphogenesis (Supplementary Tables S4 and S5). Using the genes involved in these biological processes, we made a Kyotto Encyclopedia of Genes and Genomes (KEGG) enrichment pathway analysis. Interestingly, we found that several piRNA target genes participate in important molecular pathways in cancer (Table 4).

## 3. Discussion

Studies in association with small non-coding RNAs have been widely explored in cancer research, aiming to reveal novel tumor biomarkers. Among these species of RNAs, the piRNAs are an emergent class, which need further studies to elucidate their roles in carcinogenesis [40,41,42,43]. PIWI-interacting RNAs (piRNAs) are known to be related to the maintenance of genomic integrity, and, for a long time, their expression was associated with restriction to the stem cells [24,25,27,44] and reproductive cells in mammals [33,45,46]. However, an increased number of studies have demonstrated that piRNAs are also found in somatic cells and cancer, regulating gene expression at transcriptional and post-transcriptional levels by epigenetic mechanisms [23,38,47,48]. Using deep sequencing technology to analyze the piRNA expression pattern in gastric cancer, we found 698 expressed piRNAs, confirming their presence and abundance in gastric somatic cells, and 14 of them are associated with gastric cancer.

Although piRNA expression had been previously demonstrated, its role in gastric cancer is still unclear [23,40]. Cheng et al. (2011) demonstrated the deregulation of piRNAs in gastric cancer and in vitro modulation of these isoforms leads to cell cycle arrest in gastric tumor cells [35,49]. Martinez et al. (2016) described the expression pattern of 273 of the ~20,000 known piRNAs in tumor tissues, reporting that most of the deregulated piRNAs were inserted into protein-coding regions and that some piRNA isoforms are associated with recurrence-free patient survival [38]. Recently, Lin et al. (2019) reported 50 piRNA isoforms associated with gastric cancer across the whole transcriptome, 60% of which were mapping within protein-coding regions [50]. In the present study, we compared three types of gastric tissue (NC, ADJ, and GC) and identified 14 differentially expressed piRNAs.

There are not many studies concerning piRNA expression in cancer, especially in gastric tumors [23,50,51]. However, one of the DE piRNAs found in the present study, piR-35407, was once described as down-expressed in breast cancer [36]. In gastric cancer, this piRNA was found overexpressed in comparison to NC and ADJ, but no differences in its expression were found between GC and ADJ. Most of the DE piRNAs described here were not yet described as cancer-related in the literature. This may be due to our study design, which was the first to include the three types of gastric tissue. 

Considering that epigenetic alterations could potentially be detected in the early stages of carcinogenesis [9,40,52], our analysis, comparing the NC and ADJ tissues, allows for better comprehension of tumor progression and highlights alterations that could be used as prognostic biomarkers. Although a few studies have demonstrated some discrepancies of piRNA expression between blood and cancer tissues, their potential as cancer biomarkers is remarkable [43,53]. In this study, we found eight possible piRNAs and some that are exclusive to GC and ADJ, such as piR-31355* (Figure 2B and Figure 3B).

When searching for biomarkers, it is important to consider that cancer is a complex disease that involves epigenetic microenvironment factors and somatic and hereditary mutations. A few studies have demonstrated that, besides their normal histological features, the adjacent tissue, which is the tissue surrounding the tumor, presents molecular alterations, which are very similar to those observed in cancer tissues, suggesting the presence of a field-effect [54,55]. Here, using next generation sequencing (NGS) data, we found three piRNAs that suggest the field effect in gastric cancer (piR-48966, piR-49145, and piR-31335). Their expressions between ADJ and GC are different in comparison to NC, but are similar between each other, indicating that piRNA expression levels are aberrant in adjacent-tissue samples. We further confirmed the involvement of piR-48966 and piR-31335 in the field-effect by qRT-PCR. As the literature observes [56,57,58], the field-effect has crucial implications in research and clinical practices as it impacts the matching adjacent-to-tumor tissue in such a way that it cannot be considered as a normal tissue. Corroborating our study, similar findings were observed in investigations involving other non-coding RNAs [9,12,52]. The combination of these data with our results may provide further knowledge about gastric carcinogenic transformation, which may be used as an informative tool to improve clinical practice.

Although piRNA deregulation was described in the literature, its impact in tumor progression remains poorly understood due to the lack of functional annotation data [23]. In an attempt to better comprehend the piRNA functions, we aligned mature piRNAs and human genome sequences and found some potential target genes. Among these, we identified eight possible cancer-related genes, which may be regulated by five of the eight DE piRNAs, and among those eight genes, FGFR2 and PEAK1 were described as cancer driver genes [40] (Table 2). Interestingly, we found a negative correlation between PIKFYVE and piR-31355* in healthy tissues. This gene was described as being related to cellular homeostasis and migration by regulating phosphatidylinositol 5-monophosphate (PtdIns5P), which was associated with the expression of oncogenes [59,60]. 

Several studies have shown that the piRNA biogenesis can be reactivated in somatic and cancerous cells, dysregulating their expression, resulting in downregulation of tumor suppressor genes and overexpression of oncogenes at the post-transcription level [43,61,62]. It was also demonstrated that piRNAs may act as mRNAs regulators, as well as the miRNAs, by repressing mRNAs via imperfect base-pairing [36,42,63]. This approach allowed us to find a list of genes (Supplementary Tables S2–S5) that were enriched in cancer-related pathways, such as cellular differentiation, cellular adhesion, and apoptosis.

Interestingly, the DE piRNA targets found in the comparison between ADJ and GC are genes involved in cellular morphogenesis and structure, which reflect the normal histological features of the ADJ tissue and the dedifferentiation typically found in tumor cells.

A group of 18 target genes was enriched in four signaling pathways in cancer (Table 4). Two of them, cellular adhesion and expression of cell adhesion molecules (CAMs), are crucial processes to improve metastasis and cellular inflammation [59,64,65]. Among the DE piRNAs, piR-36339* had the highest number of gene targets. Its overexpression in GC in comparison to NC, hypothetically, indicates that this piRNA may silence CAMs and may become a potential biomarker of metastasis. In this way, we expect that CAM piRNA regulators are overexpressed, also in ADJ tissue.

According to previous studies, piRNAs may be reactivated during the process of tumorigenesis. Fagegaltier et al. (2016) demonstrated that Hippo signaling inhibition may contribute to this activation [47]. Here, we identified six genes from this pathway as piRNA potential targets (Table 4), suggesting that the aberrant expression of piRNAs in cancer may silence the genes involved in this pathway, indicating a possible complex process of regulation in loop.

Our functional analysis also revealed that the deregulated piRNAs may affect the TGF-beta pathway, specifically by targeting the genes *BMP5*, *BMPR1A*, *SMAD2*, and *TGFB3*. This pathway plays key roles in carcinogenesis and its deregulation is involved in a cascade of aberrant events that trigger cell growth and proliferation pathways, such as JNK and ERK, contributing to tumor progression, cell invasion, and metastasis [66]. It also promotes epithelial-mesenchymal transition (EMT) in late stages of GC, enhancing tumor cell stemness, cell invasion, chemo-resistance, and metastasis with CAMs [67,68].

Recent studies have shown piRNAs expression in many diseases, suggesting their potential as biomarkers. Here, we report the differential expression of piRNAs in non-cancer, gastric cancer, and adjacent tissues, corroborating studies that suggest the field effect in GC. Our functional analyses revealed the potential involvement of piRNAs in key processes of early gastric carcinogenesis, tumor progression, and metastasis. Particularly, our study suggests a set of three piRNAs that may become potential biomarkers of gastric cancer. However, several aspects of the biology of piRNAs in gastric cancer remain to be investigated. Hence, further studies are needed not only to improve our knowledge about the piRNA’s epigenetic mechanisms of gene regulation, but also to provide their validation as biomarkers in gastric cancer.

## 4. Materials and Methods

### 4.1. Biological Material and Clinical Data Collection

A total of 84 samples of the stomach antrum region were collected: i) 28 samples without cancer that were collected during the procedure of upper digestive endoscopy (non-cancer(NC) samples); ii) 28 samples of gastric cancer tissue (GC), and iii) 28 samples of matched tumor-adjacent gastric tissue histopathologically diagnosed as non-tumoral (ADJ). Sample sizing was according to patient availability.

All samples were obtained prior to antibiotic, chemotherapeutic, and/or radiotherapeutic treatments from patients from the João de Barros Barreto University Hospital (HUJBB), located in the city of Belém (Pará, Brazil). Immediately after collection, all samples were frozen and stored in liquid nitrogen until analysis.

All cases investigated in this study were reviewed and confirmed by a pathologist. It should be noted that the histopathological analysis of the tumor fragments was performed according to Lauren’s classification (30).

All research procedures were carried out in accordance with the Declaration of Helsinki and the Nuremberg Code, in compliance with the National Health Council’s Research Guidelines Involving Human Beings (Res. CNS 466/12), and approved (protocol number CAAE 43961215.9.0000.5634). Informed consent was obtained from all individual participants included.

### 4.2. Total RNA Extraction

Total RNA was extracted using TRIzol^®^ Reagent (Thermo Fisher Scientific, Waltham, MA, USA). After isolation, total RNA was stored at −80 °C until further analysis. Total RNA amount and integrity were determined using the Qubit 2.0 (Life Technologies, Foster City, CA, USA) and Agilent 2200 TapeStation (Agilent Technologies, Santa Clara, CA, USA), according to manufacturer’s specification. 

### 4.3. piRNA Library Preparation

Library preparation was performed using an input concentration of 1/5 µL per sample. We synthesized 24 libraries (8 NC, 8 GC, and 8 ADJ samples) using a TruSeq Small RNA Library Preparation kit (Illumina^®^, San Diego, CA, USA), according to the manufacturer’s instructions. The RNA library pool was quantified on ABI 7500 equipment (Applied Biosystem, Foster City, CA, USA) by using a KAPA Library Quantification Kit (KAPA Biosystems, Woburn, MA, USA). The libraries were sequenced on the MiSeq Sequencing System (Illumina^®^, San Diego, CA, USA) using the MiSeq Reagent Kit v3 150 cycle (Illumina^®^, San Diego, CA, USA).

### 4.4. NGS Reads Alignment and Quality Control

After sequencing, the sequencing adapters were removed using trimmomatic software [69] and the reads were trimmed and quality filtered (QV > 25). STAR Aligner (v. 2.4.0.1) [70] was used to map the reads to human genomes (v. hg19).

Once the reads were aligned, the HTseq-count [71] was used to quantify sncRNA expression. mirBase [72] and piRbase annotations [73] were used to estimate microRNA and piRNA expression, respectively. For the identification of other transcripts, we used the ENSEMBL annotation (www.ensembl.org). Before piRNA quantitation, we grouped the co-localized piRNAs to avoid ambiguous counting (Supplementary Table S6).

### 4.5. Differential Expression Analysis

To identify the differentially expressed (DE) piRNAs, three comparisons were made: i) gastric cancer (CG) vs. non-cancer (NC) samples, ii) matched tumor-adjacent gastric tissue (ADJ) vs. NC samples, and iii) GC vs. ADJ samples. For these analyses, raw read count (read counts ≥10) was performed with DESeq2 library [74].

piRNAs satisfying the following criteria were tagged as differentially expressed, |fold-change| > 3 and *p*-value < 0.05. For graphical analysis of piRNAs, expression data were normalized to reads per kilobase million (RPKM). The area under the curve (AUC > 0.85) from the receiver operating characteristic (ROC) analysis was used to identify potential biomarkers. A discriminant analysis of principal components (DAPC) was made to identify clusters of expressed piRNAs between the tissues. All statistical analyses were performed in an R platform (version 3.2.1). 

### 4.6. Validation of DE piRNAs by qRT-PCR

We further validated piR-48966*, piR-31355*, and piR-49145 expression by qRT-PCR in 60 tissue samples (20 NC, 20 GC, and 20 ADJ). For the detection of piRNA levels, the TaqMan MicroRNA Reverse Transcription Kit (Thermo Scientific) was used to generate cDNA, according to the manufacturer’s instructions. Real-time PCR was performed using a TaqMan Universal Master Mix II (Thermo Scientific) and custom probes (Custom TaqMan Small RNA Assays - Thermo Scientific). The probe sequences were: 5′-TGGGGCGAAGCTACCATC-3′ (piR-48966*), 5′-GGCCGTGATCGTATAGTGGTTAGTAC-3′ (pirR31355), and 5′-TGAGGTAGTAGGTTGTATGGTTTAG-3′ (piR49145). We used RNU6B as endogenous to allow the comparative Ct method. Assays were performed in triplicate on ABI 7500 (Applied Biosystems, Foster City, CA, USA).

Statistical analysis was performed by the Shapiro–Wilk test (Supplementary Table S7) followed by Kruskal–Wallis tests for multiple comparison and the Wilcoxon Mann–Whitney test to paired comparisons. The results were adjusted for multiple comparisons using Bonferroni’s correction. Adjusted *p*-values of <0.05 were considered statistically significant.

### 4.7. Identification of DE piRNA Target RNAs

Potential RNA targets of differentially expressed piRNAs were identified by using mature sequences of piRNAs as a query to run a BLAST search. Results that exhibited at least 13 contiguous nucleotide complementary matches were considered potential targets of piRNA regulation [63]. We also correlated the expression of the DE piRNAs to the genes by analyzing 64 paired tissues from the ENCODE database (Supplementary Table S8).

PiRNA target genes were identified by predicting the complementarity sequence between each piRNA and the 5′-UTRs, coding sequences (CDSs), or 3′-UTRs of all known human mRNAs (RefSeq gene annotations, Human genome assembly, GRCh38/ hg18), with miRanda [v3.3a], applying a stringent alignment score (sc ≥170), energy threshold (free energy ≤ −30.0 kcal/mol) (27), and at least 80% of complementarity. Functional analysis was performed using DAVID Bioinformatics Resources [v 6.8] [75,76] to identify all biological processes significantly associated (*p* value ≤ 0.05) with piRNA-targeted mRNAs. 

### 4.8. Dataset Availability

The datasets used during the present study are available at European Nucleotide Archive (EBI-ENA) under accession number PRJEB27213.

## Figures and Tables

**Table 1:** Differentially expressed piRNAs (adjusted *p*-value < 0.05; |fold-change| > 3) in each comparison.

Differentially Expressed piRNAs	GC vs NC		AD vs NC		GC vs AD	
	*log2Fold Change*	*pAdj*	*log2Fold Change*	*pAdj*	*log2Fold Change*	*pAdj*
piR-48966 *	2.26	3.49 × 10^−3^	l.78	0.015		
piR-49145	2.18	7 × 10^−3^	3.01	2 × 10^−8^		
piR-31355 *	2.24	2.42 × 10^−5^	2.72	7.09 × 10^−6^		
piR-33864	2.59	0.01				
piR-36246	2.49	9.70 × 10^−3^				
piR-36339 *	2.04	0.02				
piR-36378	2.02	0.01			2.34	0.03
piR-33534 *					3.11	3.14 × 10^−4^
piR-39060 *			−4.27	1.92 × 10^−17^	3.21	3.62 × 10^−4^
piR-32678			−2.23	5.64 × 10^−6^		
piR-34373 *			1.75	2.28 × 10^−6^		
piR-34378 *			1.99	2.26 × 10^−5^		
piR-35407			2.25	0.03		
piR-44984			1.83	0.02		

(−) indicates down expression; (*) co-localized piRNAs, according to piRbase’s annotation. See Supplementary Table S2 for the complete list of differentially expressed (DE) co-localized piRNAs found.

**Table 2:** Complementarity between the DE piRNAs and some cancer-related genes at the transcriptional level.

piRNA	Target Gene	# of Complementary Sites	Energy to Most Probably Complementary Site	Score to Most Probably Complementary Site
piR-48966 *	*LYN^†^*	48	−24.43	219
piR-31355 *	*PIKFYVE*	7	−22.92	147
piR-33864	*BIN1*	16	−25.37	162
	*FGFR2 ^†^*	33	−23.60	173
piR-33534	*FBXO31*	1	−30.08	147
piR-39060	*PRUNE1*	25	−20.67	166
	*PEAK1 ^†^*	123	−35.75	175
	*CRLF3*	34	−26.07	161

(*) Co-localized piRNAs, according to piRbase’s annotation; (†) cancer driver genes [40].

**Table 3:** Complementarity of DE piRNAs with mRNAs of human genes at the post-transcriptional level.

piRNA	No. of Complementary Sites	No. of Genes	Maximum Score	Minimum Energy	Maximum Complementarity
piR-31355 *	169	63	230	−49.6	100
piR-32678	4	3	185	−36.9	85.7
piR-33534 *	57	23	190	−34.45	85.2
piR-33864	11	8	178	−33.27	84.0
piR-34373 *	10	2	192	−31.08	92.0
piR-34378 *	460	119	204	−38.75	86.2
piR-35407	286	146	200	−48.77	81.5
piR-36246	33	16	186	−40.18	88.0
piR-36339 *	409	157	195	−43.17	84.6
piR-36378	82	46	231	−55.10	100
piR-39060	33	19	187	−35.14	81.5
piR-44984	59	42	235	−55.40	100
piR-48966 *	191	101	198	−35.30	88
piR-49145	444	252	192	−36.46	88.9

(*) Co-localized piRNAs, according to piRbase’s annotation. See Supplementary Table S1 for the complete list of DE co-localized piRNAs found.

**Table 4:** Kyotto Encyclopedia of Genes and Genomes (KEGG) enrichment pathway to target genes of piRNAs.

Molecular Pathway	Observed Gene Count	FDR	Genes
Cell adhesion molecules (CAMs)	9	9.77 × 10^6^	*CD276, CD8A, CD99, CLDN19, HLA-G, MAG, NCAM1, NLGN4Y, SIGLEC1*
Renal cell carcinoma	4	0.0248	*HIF1A, PAK6, PTPN11, TGFB3*
TGF-beta signaling pathway	4	0.0318	*BMP5, BMPR1A, SMAD2, TGFB3*
Hippo signaling pathway	6	0.0338	*BMP5, BMPR1A, SMAD2, TGFB3, WNT4, BMPR2*

## Supplementary Materials

Supplementary materials can be found at https://www.mdpi.com/1422-0067/21/20/7656/s1.

## Abbreviations

ADJ	Samples of matched non-tumoral tumor-adjacent gastric tissue
AUC	Area Under the Curve (ROC analysis)
CAMs	Cell Adhesion Molecules
DE	Differentially Expressed
EMT	Epithelial-Mesenchymal Transition
GC	Gastric Cancer
KEGG	Kyotto Encyclopedia of Genes and Genomes
miRNA	MicroRNA
NC	Non-Cancer tissue
NGS	Next Generation Sequencing
piRNA	Piwi-Interacting RNA
pi-RISC	piRNA-mediated RNA silencing complex
qRT-PCR	Quantitative Real-Time PCR
sncRNA	Small non-coding RNA
