# Supplementary material for: Global Analyses of Expressed Piwi-Interacting RNAs in Gastric Cancer
Source: Int J Mol Sci. 2020 Oct 16;21(20):7656. doi: 10.3390/ijms21207656 (PMC7593925; doi:10.3390/ijms21207656)
Supplement: Supplementary file 1 [file ijms-21-07656-s001.zip › Supplementary Materials for conversion/ijms-877694_supplementary_figures for proof.pdf]

Supplementary figures

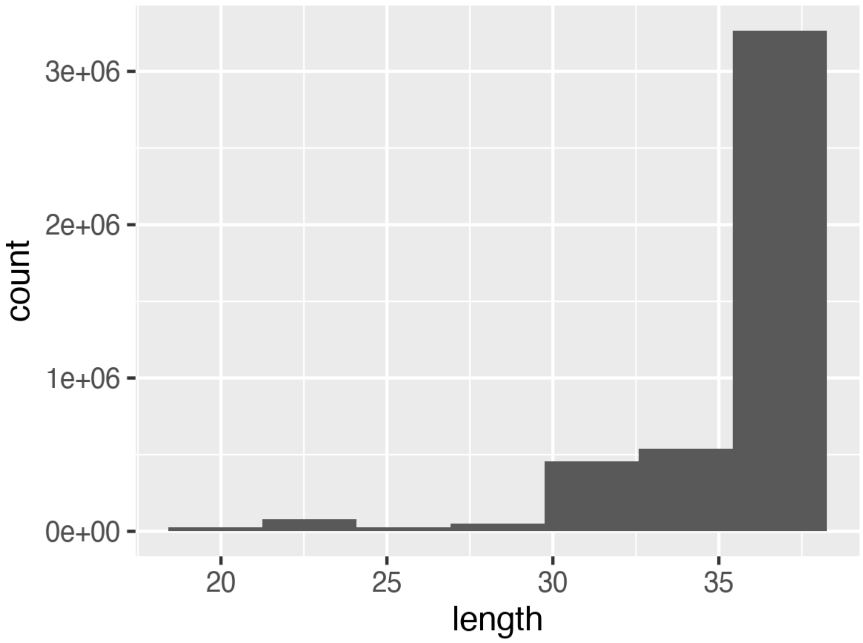

Figure S1. Distribution by size of piRNAs identified in small RNA-seq of 24 gastric tissues.

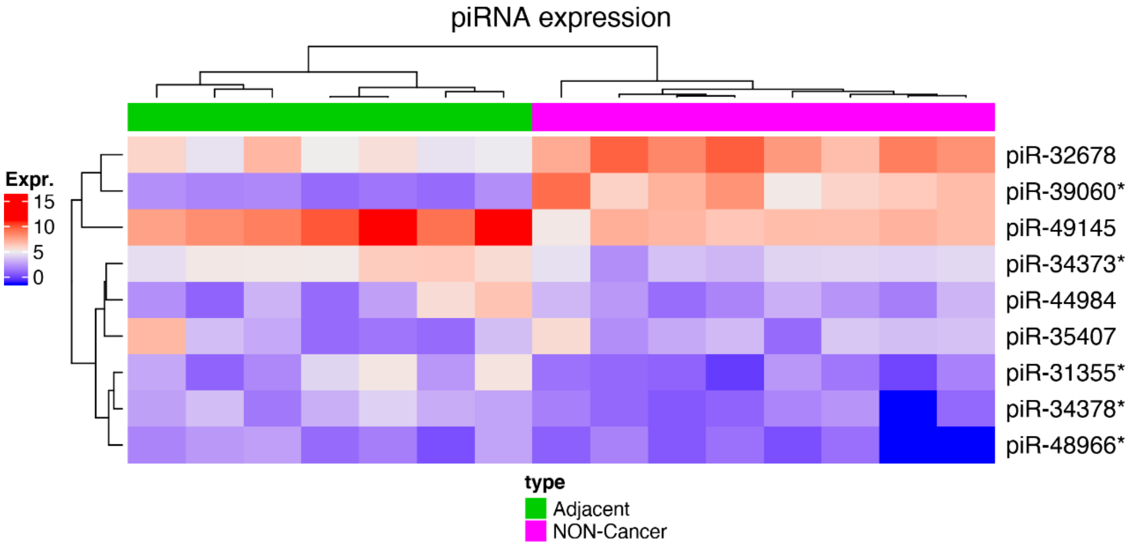

Figure S2. Heatmap of differentially expressed piRNAs bettewn seven tumor-adjacent gastric tissue and eight non-cancer tissue.

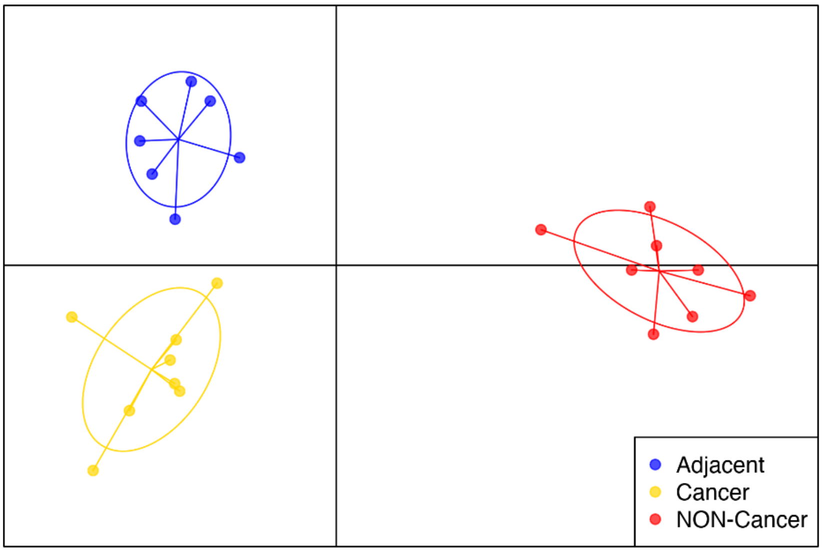

Figure S3. Comparison of the differentially expressed piRNAs (|fold- change| > 3 and *p*-value < 0.05) among all three analysis. DAPC plot clustering samples (ADJ.100927 sample excluded) based on differentially expressed piRNAs.

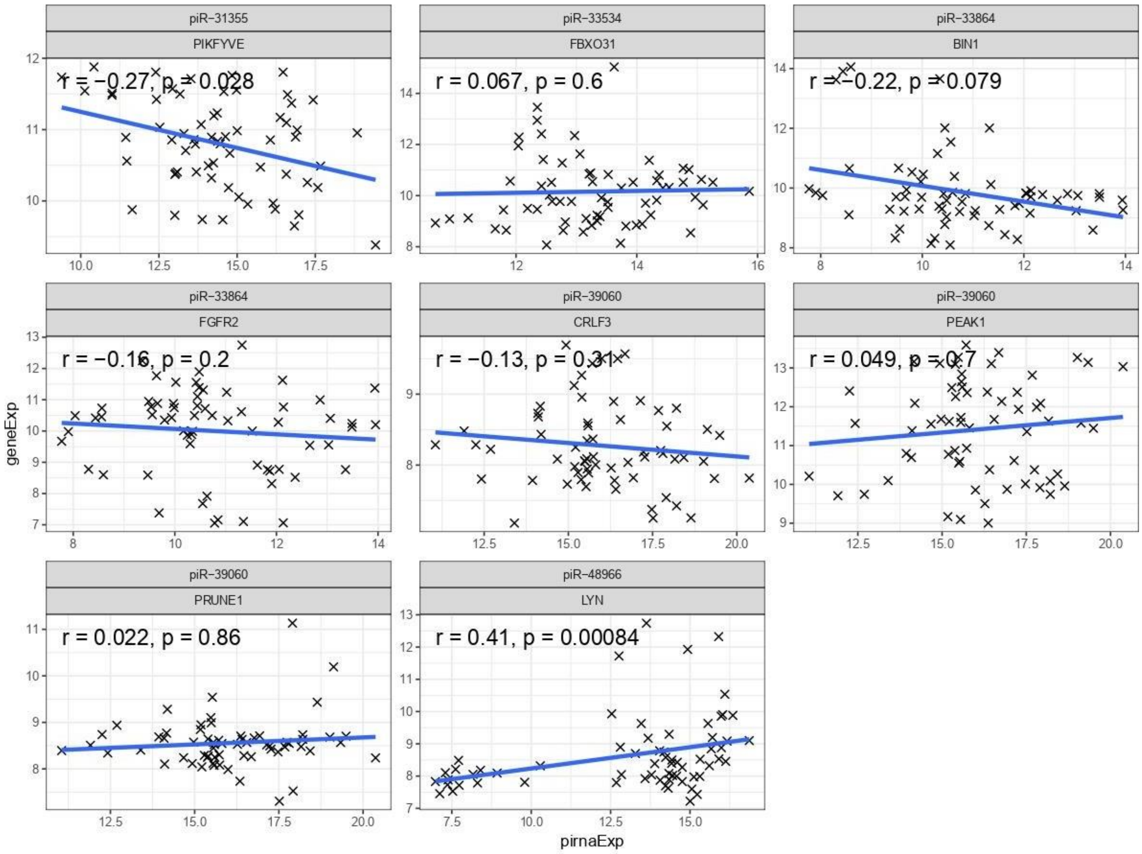

Figure S4. Correlation of the expression of DE piRNAs and their possible target genes using RNA-Seq and small RNA-Seq data from 64 paired tissues from ENCODE.
