# Supplementary material for: Global Analyses of Expressed Piwi-Interacting RNAs in Gastric Cancer
Source: Int J Mol Sci. 2020 Oct 16;21(20):7656. doi: 10.3390/ijms21207656 (PMC7593925; doi:10.3390/ijms21207656)
Supplement: Supplementary file 1 [file ijms-21-07656-s001.zip › Supplementary Materials for conversion/ijms-877694_supplementary_table _2.docx]

**Table S2.** Co-localized piRNAs DE according to piRbase’s annotation.

| piRNAs Differentally Expressed | Co-localized piRNAs | GenBank Accession | piRbase's annotation |
| --- | --- | --- | --- |
| piR-48966* | piR-48966 | DQ580854 | piR-hsa-11080 |
|  | piR-31080 | DQ570968 | piR-hsa-1219 |
|  | piR-36233 | DQ598167 | piR-hsa-28382 |
| piR-49145 | piR-49145 | DQ581033 | piR-hsa-11362 |
| piR-31355* | piR-31355 | DQ571243 | piR-hsa-1580 |
|  | piR-36038 | DQ597972 | piR-hsa-28187 |
|  | piR-36039 | DQ597973 | piR-hsa-28188 |
|  | piR-36040 | DQ597974 | piR-hsa-28189 |
|  | piR-36041 | DQ597975 | piR-hsa-28190 |
| piR-33864 | piR-33864 | DQ593752 | piR-hsa-24000 |
| piR-36246 | piR-36246 | DQ598180 | piR-hsa-28395 |
| piR-36339* | piR-36339 | DQ598273 | piR-hsa-28488 |
|  | piR-44715 | DQ576603 | piR-hsa-4945 |
|  | piR-44716 | DQ576604 | piR-hsa-4946 |
| piR-36378 | piR-36378 | DQ598312 | piR-hsa-28527 |
| piR-33534* | piR-33534* | DQ593422 | piR-hsa-23670 |
|  | piR-33535 | DQ593423 | piR-hsa-23671 |
|  | piR-33536 | DQ593424 | piR-hsa-23672 |
|  | NA | NA | piR-hsa-32492 |
| piR-39060* | piR-39060 | DQ600994 | piR-hsa-31280 |
|  | NA | NA | piR-hsa-32299 |
| piR-32678 | piR-32678 | DQ582566 | piR-hsa-12789 |
| piR-34373* | piR-34373 | DQ596307 | piR-hsa-26523 |
|  | piR-34374 | DQ596308 | piR-hsa-26524 |
|  | piR-34375 | DQ596309 | piR-hsa-26525 |
|  | piR-30504 | DQ570392 | piR-hsa-672 |
| piR-34378* | piR-34378 | DQ596312 | piR-hsa-26528 |
|  | piR-34379 | DQ596313 | piR-hsa-26529 |
|  | piR-34380 | DQ596314 | piR-hsa-26530 |
| piR-35407 | piR-35407 | DQ597341 | piR-hsa-27616 |
| piR-44984 | piR-44984 | DQ576872 | piR-hsa-7193 |

(*) indicates co-localized piRNAs.

(NA) piRNAs noted in piRBase's annotation, but not present in GenBank.
