# Supplementary material for: Global Analyses of Expressed Piwi-Interacting RNAs in Gastric Cancer
Source: Int J Mol Sci. 2020 Oct 16;21(20):7656. doi: 10.3390/ijms21207656 (PMC7593925; doi:10.3390/ijms21207656)
Supplement: Supplementary file 1 [file ijms-21-07656-s001.zip › Supplementary Materials for conversion/ijms-877694_supplementary_table _3.docx]

**Table S3.** Alignment between DE piRNAs and coding genes sequences.

| **Differentially expressed piRNAs** | **Genes** |
| --- | --- |
| piR-48966 | *DOCK10* |
|  | *CHM1 (CNMD)* |
|  | *ERLIN1* |
|  | *PHIP* |
|  | *LYN* |
| piR-49145 | *CHM1 (CNMD)* |
|  | *MIRLET7C* |
| piR-31355 | *PIKFYVE* |
|  | *STX8* |
|  | *RDX* |
|  | *PTPRT* |
| piR-33864 | *BIN1* |
|  | *FGFR2* |
| piR-36246 | *ATP5A1* |
|  | *DCAF4* |
| piR-36339 | *BHLH (TWIST1)* |
|  | *ALOXE3* |
|  | *STK32B* |
|  | *PDHX* |
| piR-36378 | *FBXO31* |
| piR-33534 | *NBEA* |
|  | *CECR2* |
| piR-39060* | *PRUNE1* |
|  | *FCGR2A* |
|  | *PEAK1* |
|  | *CRLF3* |
| piR-32678 | *ROCK1* |
| piR-34373* | *LUZP2* |
|  | *LCOR* |
| piR-35407 | *ERCC1* |
|  | *PARVB* |
|  | *PGF* |
|  | *BMPR2* |
| piR-44984 | *DOCK10* |
