# Supplementary material for: Global Analyses of Expressed Piwi-Interacting RNAs in Gastric Cancer
Source: Int J Mol Sci. 2020 Oct 16;21(20):7656. doi: 10.3390/ijms21207656 (PMC7593925; doi:10.3390/ijms21207656)
Supplement: Supplementary file 1 [file ijms-21-07656-s001.zip › Supplementary Materials for conversion/ijms-877694_supplementary_table _4.docx]

**Table S4.** Gene ontology analysis of all biological processes involving the 549 mRNAs targeted by seven piRNAs differentially expressed in non-cancer and gastric cancer tissue samples identified in this study. Just functional annotation with p value ≤ 0.05 are shown.

| **Biological Process** | **Genes** | **No. of genes** | ***p* value** |
| --- | --- | --- | --- |
| Cell adhesion | CD99, SLC11A1, SDK2, LRP6, EPHA8, CELSR1, GNAS, SIGLEC1, NUDC, CCDC80, CDHR5, TRIOBP,CD99L2, SRPX2, CD276, LILRB1, SIGLEC5, MAG, ZC3HAV1, CEP41, LRRC59, TECTA, EHD1, BMP5, CLINT1, PCDH12, PCDH17, SLC46A2, HSPH1, TNFSF9, MACF1, NCAN, SNX9, LILRB2, AOC3, EMILIN2, MPRIP, PLXNC1, UBAP2, SDCBP, PAK6, BCL10, ITGA10, NECTIN4, RORC, MUC4, NLGN4X, NCAM1, PCDH15, CDH8, ASTN1, UTRN, CNTNAP4, CD8A, PKNOX1, WNT4, DISC1, CLASP2, CLDN19, NLGN4Y, DCHS1, MAPK7, AXL, CX3CR1, SUSD5, IL20RB, KLC2, PTPN11, EGR3, ZFPM1, MYADM, GAS6, TACSTD2, CSF1, ROBO2, ATP4B, VWC2, PTPRT, EPHB4, DMD, HLA-G, ITGA1, PCDHAC1, PCDHA10, CD24 | 85 | 0.013 |
| Positive regulation of cell differentiation | BTK, LMO3, GNAS, TRIOBP, HIF1A, NFATC2, CD276, MAG, BMPR1A, EHD1, OPRM1, BMP5, ARSB, PROC, TGFB3, TNFSF9, MACF1, LILRB2, SDCBP, DUOXA1, GATA6, SYT2, DRD2, PPARGC1B, WNT4, DISC1, GPRC5B, AXL, CX3CR1, CMKLR1, SMAD2, EGR3, MYADM, GAS6, TACSTD2, CSF1, ROBO2, PTCH1, VWC2, DMD, HLA-G, NCKIPSD, CPNE1, CD24, NEFL | 45 | 0.026 |
| Regulation of leukocyte apoptotic process | BTK, HIF1A, LILRB1, SLC46A2, BCL10, RORC, AXL, GAS6 | 8 | 0.030 |
| Positive regulation of cytokine-mediated signaling pathway | HIF1A, AXL, GAS6, CSF1, CPNE1 | 5 | 0.037 |
| Cell-cell adhesion | SLC11A1, SDK2, LRP6, CELSR1, GNAS, SIGLEC1, NUDC, CDHR5, SRPX2, CD276, LILRB1, ZC3HAV1, CEP41, LRRC59, EHD1, BMP5, CLINT1, PCDH12, PCDH17, SLC46A2, HSPH1, TNFSF9, MACF1, SNX9, LILRB2, MPRIP, UBAP2, SDCBP, PAK6, BCL10, NECTIN4, RORC, NLGN4X, PCDH15, CDH8, ASTN1, CD8A, PKNOX1, WNT4, CLDN19, NLGN4Y, DCHS1, MAPK7, IL20RB, KLC2, PTPN11, EGR3, ZFPM1, MYADM, GAS6, TACSTD2, ROBO2, PTPRT, HLA-G, PCDHAC1, PCDHA10, CD24 | 57 | 0.042 |
