# Supplementary material for: Global Analyses of Expressed Piwi-Interacting RNAs in Gastric Cancer
Source: Int J Mol Sci. 2020 Oct 16;21(20):7656. doi: 10.3390/ijms21207656 (PMC7593925; doi:10.3390/ijms21207656)
Supplement: Supplementary file 1 [file ijms-21-07656-s001.zip › Supplementary Materials for conversion/ijms-877694_supplementary_table _5.docx]

**Table S5.** Gene ontology analysis of all biological processes involving the 608 mRNAs targeted by seven piRNAs differentially expressed in non-cancer and adjacent to gastric cancer tissue samples identified in this study. Just functional annotation with *p* value ≤ 0.05 are shown.

| **Biological Process** | **Genes** | **No of genes** | ***p* value** |
| --- | --- | --- | --- |
| Regulation of ras protein signal transduction | ARHGEF5, GBF1, RASGRF2, ARFGEF2, ARHGEF11, PSD2, FARP1, DGKI, ABCA1, SCAI, ABRA, CSF1, ECT2L, ARHGEF33, ARHGEF38 | 15 | 0.027 |
| Cell adhesion | SLC11A1, TRO, SDK2, RAB27A, IGSF9, GNAS, CCDC80, BMP7, SRPX2, CD276, TJP1, IL7, LILRB1, SIGLEC5, MAG, CEP41, CDH23, TECTA, EHD1, NRXN2, CORO1C, KRT18, BMP5, CLINT1, PCDH17, NKX23, HSPH1, TGFBI, TNFSF9, LRFN3, LILRB2, AOC3, EMILIN2, MPRIP, DSC2, SDCBP, PAK6, CDH24, BCL10, NECTIN4, RORC, MEGF10, TNFRSF21, NLGN4X, CDH22, PCDH15, CDH8, ASTN1, UTRN, CNTNAP4, CDH12, WNT4, DISC1, CLDN19, PCDH19, VWA2, DCHS1, AXL, TNXB, SUSD5, SELP, IL20RB, KLC2, CDH4, PTPN11, ZFPM1, TACSTD2, CSF1, ATP4B, PPARA, VWC2, RELN, PTPRT, EPHB4, COL13A1, DMD, TGM2, BTNL2, PCDHA7, PCDHA1, ITGA1, PCDHA10 | 82 | 0.010 |
| Homophilic cell adhesion via plasma membrane adhesion molecules | TRO, SDK2, IGSF9, CDH23, PCDH17, DSC2, CDH24, NECTIN4, CDH22, PCDH15, CDH8, CDH12, PCDH19, DCHS1, CDH4, PTPRT, PCDHA7, PCDHA1, PCDHA10, | 19 | 0.035 |
| Cell-cell adhesion via plasma-membrane adhesion molecules | TRO, SDK2, IGSF9, CDH23, PCDH17, DSC2, CDH24, NECTIN4, CDH22, PCDH15, CDH8, CDH12, CLDN19, PCDH19, DCHS1, SELP, CDH4, PTPRT, PCDHA7, PCDHA1, PCDHA10, | 21 | 0.048 |
